# Psychological Stress, Its Reduction, and Long-Term Consequences: What Studies with Laboratory Animals Might Teach Us about Life in the Dog Shelter

**DOI:** 10.3390/ani10112061

**Published:** 2020-11-07

**Authors:** Michael B. Hennessy, Regina M. Willen, Patricia A. Schiml

**Affiliations:** 1 Department of Psychology, Wright State University, Dayton, OH 45435, USA; patricia.schiml@wright.edu; 2 HaloK9Behavior, Xenia, OH 45385, USA

**Keywords:** shelter dog, stress, hypothalamic–pituitary–adrenal, cortisol, glucocorticoid, social buffering, enrichment, early-life stress, individual differences, animal welfare

## Abstract

**Simple Summary:**

Experiments in laboratory animals have provided the basis for studies of stress, its reduction, and its long-term consequences in shelter dogs. Stressors often used in laboratory experiments, such as uncontrollable noise and novelty, are also inherent in shelters where they produce similar physiological reactions, including elevations of circulating levels of glucocorticoid stress hormones. We review how experiments demonstrating a social partner can reduce glucocorticoid responses in the laboratory guided studies showing that human interaction can have similar positive effects on shelter dogs. We also describe recent work in which human interaction in a calming environment reduced aggressive responses of fearful shelter dogs in a temperament test used to determine suitability for adoption. Finally, we present evidence from the laboratory that stress can produce long-term effects on behavior (e.g., reduced socio-positive behavior) that may be due to glucocorticoids or other factors, and which may not occur until long after initial stress exposure. We suggest that the possibility of similar effects occurring in shelter dogs is a question deserving further study.

**Abstract:**

There is a long history of laboratory studies of the physiological and behavioral effects of stress, its reduction, and the later psychological and behavioral consequences of unmitigated stress responses. Many of the stressors employed in these studies approximate the experience of dogs confined in an animal shelter. We review how the laboratory literature has guided our own work in describing the reactions of dogs to shelter housing and in helping formulate means of reducing their stress responses. Consistent with the social buffering literature in other species, human interaction has emerged as a key ingredient in moderating glucocorticoid stress responses of shelter dogs. We discuss variables that appear critical for effective use of human interaction procedures in the shelter as well as potential neural mechanisms underlying the glucocorticoid-reducing effect. We also describe recent studies in which enrichment centered on human interaction has been found to reduce aggressive responses in a temperament test used to determine suitability for adoption. Finally, we suggest that a critical aspect of the laboratory stress literature that has been underappreciated in studying shelter dogs is evidence for long-term behavioral consequences—often mediated by glucocorticoids—that may not become apparent until well after initial stress exposure.

## 1. Introduction

There is a vast literature documenting the consequences of psychological stress in laboratory animals. Many of these studies are translational in that they use rats, mice, or other species as models to provide insight into how stress exposure in humans can impair emotional wellbeing and promote the development of mental as well as physical disorders. A common procedure is to expose animals to one or more stressors that are uncontrollable and often unpredictable. These may include isolation, noise, separation from companions, and confinement or restraint. These manipulations are often found to increase behaviors thought to share processes underlying human psychopathology such as anxiety, depression, or post-traumatic stress disorder, as well as to reduce some cognitive abilities [1,2,3,4,5,6,7]. Even limiting the amount of nesting material provided to lactating female rats, which then disrupts the treatment the females provide their litters, has multiple negative outcomes on the offspring at later ages [8].

Now consider the experience of a dog (or lactating bitch and pups) that suddenly find themselves confined in an animal shelter. While housing conditions vary substantially across shelters, stressors like those that contribute to serious deleterious consequences for laboratory animals are still unavoidably inherent to some degree across shelter environments. From the perspective of a laboratory that has split its efforts between basic and translational studies of psychological stress with laboratory animals and studies to measure and reduce stress and its effects in shelter dogs, the similarities between shelter conditions and laboratory paradigms designed to induce adverse emotional and behavioral outcomes are impossible to dismiss. Both shelter housing and laboratory stress paradigms induce physiological stress responses, perhaps most importantly, though not exclusively, activation of the hypothalamic–pituitary–adrenal (HPA) axis, e.g., [6,9]. Not only can HPA activation be taken as a sign of stress in shelter dogs, but the repeated or prolonged activation of the HPA system, and particularly of the glucocorticoid hormones that are the endpoint of the HPA response, may serve as a mechanism underlying many of the long-term effects of prior psychological stress [10,11].

Fortunately, basic research has also suggested means by which the impact of stressors can be minimized. Prominent among these is the process referred to as “social buffering”, or the ability of a companion to moderate physiological stress responses. Social buffering is both a basic phenomenon we have studied over the years as well as a strategy we have used to attempt to improve welfare and preclude later adverse consequences of shelter confinement. In the remaining portions of this paper, we will view our work with dogs and related studies by other investigators within the broader context of basic and translational research on stress and social buffering.

## 2. HPA Responses and Social Buffering in the Shelter

### 2.1. How Stressors Like Those in the Shelter Affect HPA Activity

It has long been known that the HPA system is especially sensitive to psychogenic stressors, that is stressors that pose no actual physical harm [12]. Events that are novel, unpredictable, or out of an individual’s control indicate that the current situation is not fully understood and can suggest that harm is likely forthcoming. How this perception activates the HPA axis is complex and still not fully understood, see [13]. However, in brief, cortical regions detecting psychogenic stressors (e.g., medial prefrontal cortex—mPFC) together with associated limbic and brain stem regions including amygdala nuclei, portions of the bed nucleus of the stria terminalis and hippocampus, as well as the nucleus of the solitary tract, relay neural signals to the region of the paraventricular nucleus of the hypothalamus (PVN). Here, they excite (or block inhibition of) neurons containing arginine vasopressin and especially corticotropin-releasing hormone (CRH), which are then released into the hypothalamic–hypophyseal portal system. Upon reaching the anterior pituitary, these peptides bind receptors to spur release of adrenocorticotropin hormone (ACTH) into the general circulation. In several minutes, ACTH reaches the adrenal cortex to trigger synthesis and release of glucocorticoid hormones, notably cortisol and corticosterone. Glucocorticoids bind with Type 1 (MR) and (particularly during stress) Type 2 (GR) receptors throughout the body, including the brain, to induce multitudinous actions on target tissues. Activation of GR receptors in the hippocampus and elsewhere also mediates negative feedback to suppress HPA activation as the stressor passes. In addition, just as some cortico-limbic inputs excite the PVN, others (from, e.g., prefrontal cortex (PFC), hippocampus) actively suppress activity of CRH and vasopressin neurons. In principle then, there are many potential routes by which social buffering can inhibit HPA activation, either by inhibiting excitatory inputs or by exciting inhibitory inputs to the PVN. Investigation of stimuli that induce HPA activation began with Selye [14]. Although social buffering is now a widely accepted concept across a number of species [15,16,17], investigation of the ability of social partners to reduce stress developed decades after Selye’s original work.

### 2.2. Brief History of Social Buffering in Nonhuman Primates

The first publication addressing social buffering of HPA activity appears to have been Hill et al.’s [18] study of surrogate-reared Old-World rhesus macaque monkeys during the first year of life. When these infants were removed from the home cage and placed into a novel cage for an hour, they had higher plasma cortisol concentrations when alone than when accompanied by their rearing surrogate. That is, the presence of the artificial mother appeared to buffer the response of the HPA axis to disturbance and exposure to novelty. If a surrogate mother could buffer cortisol responses of infants, then one would certainly expect an actual mother to be effective as well. Indeed, later studies confirmed that cortisol elevations of rhesus infants that had been handled or handled and exposed to novel surroundings were significantly reduced when in the presence of their biological mother as compared to when they were alone [19,20]. This effect of the mother’s presence was not restricted to just rhesus or other Old World monkeys. In New World squirrel monkeys, both infants [21] as well as their mothers [22] had higher plasma cortisol concentrations following disturbance if tested without the other member of the dyad than if mother and infant remained together. On the other hand, not all affiliative social companions appeared capable of buffering HPA responses. In squirrel monkey troops, some of the most amicable interactions occur among juvenile peers which avidly engage in play, and in adult females which spend much time in close proximity with one another. Yet, juveniles did not reduce the cortisol response of familiar juveniles, and adult females did not reduce the cortisol response of familiar, even preferred, adult females [23,24].

Together these results suggested there was something special about the mother–infant relationship that enabled the partners to buffer each other’s stress responses. The most obvious possibility was simply that the degree of social connection between partners was critical; specifically, that the intensity of the relationship between mother and infant was greater than the affiliation among other friendly partners. Notably, the data did not allow one to exclude other unspecified attributes that might be characteristic of only mothers and infants. However, a second New World primate, the titi monkey, provided some insight. Unlike squirrel monkeys, titi monkeys are monogamous, with adults typically spending long periods of time in quiet contact with their pair-mate. Additionally, whereas young squirrel monkeys ride on the back of their mothers, and never their fathers, titi infants ride on the backs of both parents, especially the father [25]. Moreover, in preference tests, the mother as well as the father more often chose to be near each other than to be near their infant, and infants, in turn, preferred being near their father rather than their mother [25]. This very unusual pattern of familial preferences (Table 1) permitted experimental dissociation of social attraction or relationship intensity from other characteristics specific to the mother–infant relationship. For these experiments, entire family groups were captured and then placed back into the home cage either alone or with a specific partner(s). While all family members showed a pronounced elevation of cortisol levels following capture when returned to the home cage alone, adult males and females showed significant reductions in cortisol only when returned with each other, i.e., their adult pair-mate, and not when returned with their infant [25]. Infants displayed a reduction in cortisol concentrations when returned only with their mothers, and a further significant reduction to the level seen in undisturbed infants when returned only with their fathers [26]. Thus, the likelihood of social buffering occurring corresponded perfectly to the strength of the social attraction between the partners (Table 1). Since the time these early experiments were conducted there have been numerous demonstrations of buffering in other species, some of which involve partners with no prior social relationship whatsoever, e.g., pairs of unfamiliar adult male rats; [27,28]. Yet, the intensity of the positive relationship between partners remains the best predictor of whether an individual will buffer the glucocorticoid response of a companion [17,28,29].

### 2.3. Social Buffering of HPA Responses in Dogs

With this general principle of the importance of the strength of the relationship in mind, our first study of social buffering in dogs compared the ability of a long-term conspecific kennelmate and the human caretaker in reducing glucocorticoid elevations in adult dogs. The dogs (7–9 years old), which had been maintained in littermate pairs continuously since ~8 weeks of age, were examined in a novel environment either alone, with their kennelmate, or with their life-long human caretaker. Although we had anticipated that the caretaker might have some effect, we were nevertheless struck by the differential influence of the two companions. Whereas, the passive presence of a dog’s human caretaker reduced the plasma glucocorticoid elevation to the novel environment, the dog’s sibling and long-term kennelmate was without effect [30]. This finding certainly seemed to speak to the affinity that dogs have evolved for humans over thousands of generations. In addition, the results documented a tangible effect of human presence on the stress physiology of dogs that might then be leveraged to reduce stress and, therefore, improve welfare of dogs confined in shelters.

The first step, however, was to determine how a stay in an animal shelter affected glucocorticoid levels. The initial experiments confirmed what was expected: The psychological stressors encountered upon entering an animal shelter powerfully activated the HPA axis. Circulating cortisol levels were nearly three times as high as those of pet dogs sampled in their home and remained so for 3 days before gradually waning [31]. Subsequent work indicated that cortisol concentrations did not decline to levels like those of pet dogs sampled under resting conditions until sometime after 10 days [9]. Work from other laboratories has generally found cortisol levels to be elevated at least about this length of time or longer [32,33,34,35,36,37], though variability among individual dogs is common, e.g., [35,37].

In our first attempt to buffer this response [31], dogs were taken from their kennels and had a blood sample collected. They were either petted or returned to their kennel for 20 min, and then a second blood sample was collected to estimate the effect of the petting. Initial results were disappointing, showing that petting had no overall effect. However, when in a follow-up analysis dogs in the petting group were partitioned based on the sex of the individual doing the petting, those dogs petted by a man showed an increase from the first to the second blood sample, while those petted by a woman showed no change across samples [31]. It appeared, therefore, that interaction with a woman prevented the sampling procedure required to collect the first blood sample from elevating cortisol levels obtained in the second sample, but interaction with a man had no buffering effect. This differential influence was determined to depend on the nature of the petting administered by men versus women. When men were trained to pet in the more soothing and quiet manner characteristic of the women, the men were as effective as the women in preventing the initial blood sampling procedure from elevating cortisol levels in the second sample [38]. This was the second lesson we learned about social buffering in dogs: Not only are humans particularly effective in buffering the glucocorticoid response of dogs, but seemingly subtle differences in how the human interacts with the dog can determine whether or not the HPA response is reduced. Still, however, we were only effective in reducing the response of the dogs to an additional minor stressor—the initial blood sampling—rather than mitigating the response to the shelter itself.

After a series of further unsuccessful attempts to reduce the glucocorticoid response to shelter housing, we came upon what appeared to be a third lesson about social buffering in shelter dogs; that is, in addition to how you interact, the location where you interact also is critical. In this study, we were able to secure a quiet, secluded room in the rear of the shelter that was farther away from the commotion of the housing and public areas than in any of our previous studies. Here, we found that it did not matter if a person petted, played with, or passively sat near the dogs. In all cases, plasma cortisol levels were reliably reduced when a person (woman) was present [39]. If the dog was simply isolated in the secluded room, there was no reduction in cortisol levels. In other words, the secluded room alone had no effect, but a person in the room, even sitting quietly with the dog, suppressed the cortisol response to shelter housing. Ours was not the first laboratory to find human interaction to reduce the cortisol response to shelter conditions. During the time of our unsuccessful attempts, two other laboratories had found human interaction to such buffering effects [33,36]. The forms of human interaction in these studies were more complex, involving a variety of activities in different locations, but both included some time outdoors, removed from the commotion of the shelter. In all, these studies document how social buffering in the form of human interaction can readily mitigate the physiological stress response imposed by inherent features of shelter housing. Yet, this strategy has clear limits in that the effect is temporary. When dogs are returned to their kennel, cortisol levels elevate to their pre-interaction levels within an hour [40]. A recent approach that greatly prolongs the beneficial effect of interaction is to foster shelter dogs to a private home for a night or two. In shelters in which this procedure is implemented, urinary cortisol levels are reduced throughout the fostering period, though here too cortisol concentrations elevate to pre-interaction levels when returned to the shelter environment [41].

### 2.4. Mechanism of Social Buffering of HPA Responses

**Figure 1:**
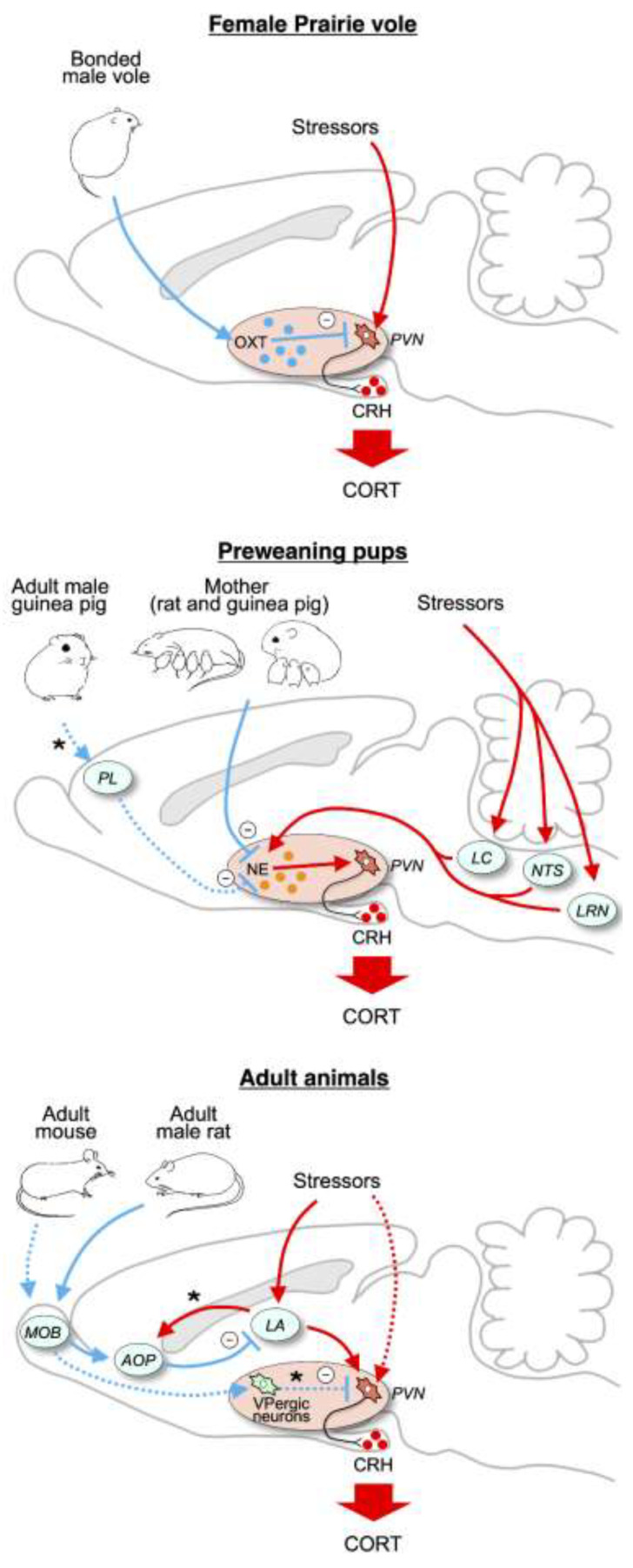
Summary of findings regarding neural circuits underlying social buffering. (**Top**) Presumed neural mechanisms underlying social buffering by mates in female prairie voles. (**Middle**) Possible neural mechanisms underlying social buffering in rodent pups. (**Bottom**) Possible neural mechanisms underlying social buffering by adult conspecifics other than mother and mates. Solid and dashed lines represent pathways proposed in each experimental model. However, the pathways do not necessarily imply direct anatomical connections. Hypothetical buffering pathways are marked by asterisks. AOP, posterior complex of the anterior olfactory nucleus; CORT, cortisol or corticosterone; CRH, corticotropic releasing hormone; LA, lateral amygdala; LRN, lateral reticular nucleus; MOB, main olfactory bulb; NE, norepinephrine; NTS, nucleus of the solitary tract; OXT, oxytocin; PI, prelimbic cortex; PVN, paraventricular nucleus of the hypothalamus; VP, vasopressin. Figure redrawn from [28].

Oxytocin appears to be the most likely mediator of social buffering of dogs’ HPA response by human interaction. Release of oxytocin both stimulates, and is stimulated by, engaging social behaviors such as gentle touch and prolonged gazing [42,43]. Furthermore, while oxytocin’s influence is much more complex than simply enhancing sociability, there is a wealth of data on how oxytocin can promote socio-positive or bonding-related behaviors [43,44,45], including those of dogs with humans or other dogs [46,47]. These effects often may be due to oxytocin reducing anxiety or wariness to engage in social activity [48]. Oxytocin can also reduce HPA activity more directly by, for instance, inhibiting excitatory input to the PVN or via inhibitory GABA interneurons connecting oxytocin neurons to corticotropin releasing hormone cells in the PVN [43,49]. Indeed, in the monogamous prairie vole, the ability of an adult male to buffer the HPA response of his female partner is inhibited by pharmacologically blocking oxytocin receptors in the PVN [50] (Figure 1, top).

However, studies in laboratory rodents have identified a number of other potential mediators that could act independent of, or in conjunction with, oxytocin. For lactating rats, evidence indicates that the mother’s buffering of HPA activity of her pups is due to inhibition of excitatory noradrenergic input to the PVN from brainstem [51]. In guinea pig pups, both the mother and an unfamiliar male can buffer HPA responses and do so through different mechanisms. The presence of the mother, even when anesthetized, reduces pups’ cortisol response during exposure to novelty [52] quite possibly again by inhibiting noradrenergic input [53]. In contrast, adult males reduce pups’ cortisol response in a novel environment when the male is awake and actively engaging the pup, but not, unlike the case for the mother, when then male is anesthetized [54]. The active male increases excitation in the pup’s PFC, which may activate known inhibitory connections to the PVN [55] (Figure 1, middle). Finally, in adult rats and mice, the ability of companions to reduce HPA responses appears due to the companion activating olfactory connections to the amygdala or directly to the PVN [56,57,58] (Figure 1, bottom). Thus, at this point it would be premature to conclude that oxytocin mediates the reduction in HPA activity in dogs interacting with humans. Furthermore, as the guinea pig data above suggest, it is even possible that different forms of human interaction (e.g., soothing touch, play) suppress HPA activity through different pathways.

## 3. Stress Effects on Behavior

### 3.1. The Challenge of Detecting Behavioral Consequences of Stress in Shelter Dogs

Stress in shelters is of concern in large part because of the possibility it will increase readily apparent behaviors such as stereotypy, hyperactivity, fearful behaviors, and continual barking that will either discourage adoption or prompt recent adopters to return their dog to the shelter, e.g., [59,60]. However, the stress endured by shelter dogs may have less conspicuous effects on behavior that are more difficult to verify experimentally. Major obstacles to the necessary experiments include the impossibility of achieving random assignment, undesirability of invasive procedures, need to accommodate shelter procedures in experimental designs, the hugely divergent past experiences of dogs who end up in shelters, and the difficulty of distinguishing effects of stress as opposed to other aspects of the shelter environment. However, while effects that are unequivocally due to stress are difficult to document, the existing literature in laboratory animals clearly points to a variety of ways that psychological stressors like those experienced in the shelter may both reduce desirable behavior and lead to later emerging behavioral and emotional repercussions, at least for some dogs. To take some examples from the broader literature, juvenile rats exposed to social instability (15 days of repeated periods of isolation followed by housing with unfamiliar conspecifics) showed lower levels of social behavior, both immediately after treatment and in adulthood [61,62]. In another study, periods of maternal separation prior to weaning led to inhibited social behavior and abnormal PFC development in juvenile female rats, whereas for males these effects did not appear until adolescence [63]. Adult mice housed individually for 8 weeks performed more poorly on several measures of cognition than did mice housed in groups [64], and rhesus macaques, whose mothers had been exposed to unpredictable noise bursts during pregnancy, scored worse than controls on measures of attention and neuromotor maturation during the first 3 weeks of life [65] and played less in adulthood [66]. Findings such as these raise concern that desirable traits, such as sociality and cognition, may be compromised as a result of the shelter experience.

**Figure 2:**
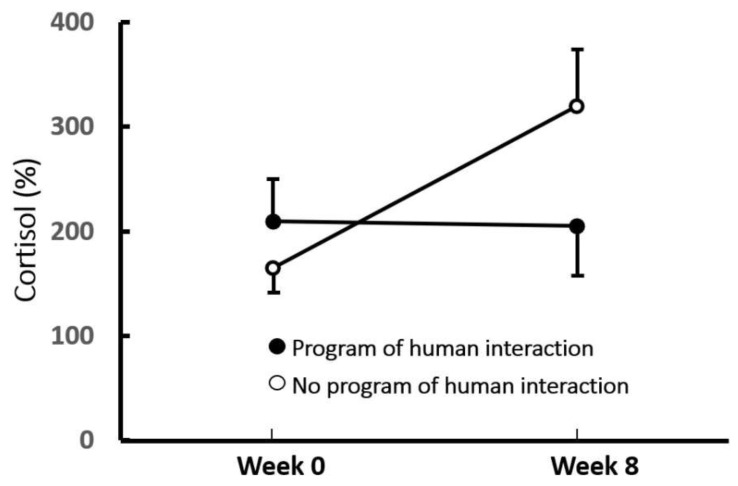
Mean per cent increase in plasma cortisol levels in response to a highly novel situation prior to and following an 8-week period in which shelter dogs either received or did not receive supplementary human interaction (5 weekly, 20-min sessions). Vertical lines represent standard errors of the means. Dogs receiving standard care, but not dogs receiving supplemental human interaction, exhibited enhanced cortisol responsiveness over the 8-week period (*p* < 0.05). Figure redrawn from Hennessy et al. [34].

Other potential long-term consequences may be more insidious. Much of the current surge in laboratory studies of lasting biobehavioral effects of stress has been driven by the increasing realization that stress at a particular life stage (primarily but not exclusively during prenatal, early postnatal, or adolescent phases) can alter the course of later development, which in humans leads to increased vulnerability to a variety of mental and physical disorders [67]. These include increased susceptibility to major depression, anxiety disorders, post-traumatic stress disorder, and schizophrenia [11,68,69,70]. Importantly, these outcomes may not emerge in humans until years later, often after a mental or physical challenge at the later age, a pattern commonly referred to as the “2-hit” model [68,70,71] because a second major stressor or “hit” is required to unmask the long-term effect. The first hit is thought to sensitize some aspect of underlying stress physiology so that the second stressor produces a larger, more prolonged, and/or unregulated stress response that then gives rise to the mental disturbance. These effects can be modeled in laboratory animals. For example, exposing adolescent mice to 12 days of unpredictable stress increased measures of anxiety-like and depressive-like behavior when the mice were placed in stressful situations 30 days later [72]. Similarly, two 3-h periods of isolation near the time of weaning increased depressive-like behavior of guinea pigs when isolated again in early adolescence [5]. If such a model applies to some extent to dogs confined in animal shelters, it implies that behavioral and welfare consequences of the stress of shelter housing may not occur until exposed to a subsequent stressor that then engages the now sensitized stress physiology, perhaps well after the dog has been adopted. One piece of evidence supporting such concern derives from an early study in our laboratory in which dogs were exposed to a highly novel stressful situation before and after 8 weeks of shelter housing. Whereas dogs that received regular sessions of human interaction throughout the 8-week period showed comparable plasma cortisol responses to the two stress sessions, those deprived of the supplemental interaction showed a significantly greater cortisol response to the second stressor [34] (Figure 2).

Glucocorticoids are, in fact, one mediator of lasting behavioral effects of stress exposure, e.g., [73,74], including on social behavior [75]. In utero effects of stress exposure appear mediated by maternal glucocorticoids acting on the fetus [76,77]. Even glucocorticoids received through the mother’s milk may influence social and cognitive development [78]. Another mediator of long-term effects is stress-induced neuroinflammatory signaling. Early-life stress upregulates central inflammatory activity in later life [79,80] and, in humans, increased inflammatory activity promotes development of stress-related disorders [81,82]. Seemingly analogous processes have been demonstrated in laboratory rodents [83,84]. Although stress in early life can affect a variety of brain regions, threat-related circuits [85] including connections between the mPFC, amygdala, and hypothalamus appear to be critical. Among the most robust effects of early-life stress is sensitization of cortico-amygdalar circuitry [86,87]. One way in which inflammatory signaling appears to promote human psychopathology is by enhancing amygdala activity, which then appears to further increase inflammation, creating a positive feedback loop promoting greater susceptibility to stress-related disorders [86,88]. Moreover, increased amygdala activity that escapes regulatory control by the PFC can then affect hypothalamic control of the HPA axis and sympathetic nervous system [89] to further promote development of stress-related pathologies [90,91,92,93].

One would not expect all dogs to be equally susceptible to the stress of shelter housing. To the extent that outcomes such as those outlined above pertain to shelter dogs, young dogs or the unborn fetuses of pregnant bitches would be most vulnerable. Further, as others have emphasized, e.g., [94], even in dogs of the same age, we should expect substantial individual differences in stress responsiveness and vulnerability. Due to a combination of experience and temperament, some dogs react much more strongly than others to admittance to a shelter. One form of enhanced reaction is aggression.

### 3.2. Reduction in Fear-Induced Aggression in the Shelter

Dogs exhibit a variety of initial reactions upon entering a shelter. While most show signs of fear, for some, the fear is extreme. These dogs may tremble, cower in the back of the kennel, and keep their tail tucked firmly between their legs. They may also show signs of fear-induced aggression [95,96], a situationally dependent form of aggression that occurs in some individuals when fear is high, and escape is thwarted. As this aggression only occurs when dogs are severely frightened, such dogs may be excellent candidates for adoption as a pet in a typical home, but in the shelter they are often in great peril. With shelters understandably concerned about the injuries and damage to the shelter’s reputation as a source of quality pets that an aggressive dog might cause, preventing dangerous dogs from being adopted becomes a priority. Some form of a “temperament test” is often used for this purpose. The SAFER^®^ (hereafter SAFER) is one such instrument. Though designed to be but one of several sources of information used to determine suitability for adoption [97], busy shelters may rely solely on the outcome of the test, or a modified version of it, to determine the fate of their confined dogs. If the test is administered a few days after entry to the shelter—before initial fear may have a chance to abate—dogs exhibiting fear-induced aggression are likely to fail and be euthanized rather than adopted.

Both our own work and that of others, e.g., [98,99], suggested that some form of human contact might help reduce the fear and aggression, and ultimately the euthanasia of such dogs. Accordingly, the second author initiated an enrichment program centered around responsive human interaction for fearfully aggressive dogs at a local shelter. The program appeared successful anecdotally and so prompted an experimental evaluation of its effectiveness [100]. This enrichment was provided in a secluded room as in our earlier work [40]. Dogs also had access to toys and were given small treats. In addition, oil of lavender was misted into the room and classical music was played softly in the background in light of the reported calming effect of these forms of stimulation [101,102]. Only dogs that exhibited signs of both a high level of fear and aggression were enrolled. Enrichment was conducted by the second author, a board-certified Associate Applied Animal Behavior Specialist with extensive experience working with shelter dogs. Dogs in a treatment group received the enrichment for 15 min, twice a day, for 5–7 days. Control dogs received normal shelter care. The day following the final treatment, or on the same average day in the shelter for controls, dogs were administered the modified version of the SAFER used by this shelter, administered by shelter staff as per shelter operating procedure. This version of the test assessed the dog’s reaction to eye contact, sensitivity to touch, movement and sound during play, response to having its paw squeezed and having its food bowl manipulated during eating, and the presence of another dog. The staff were unaware that performance on the SAFER was an outcome that we measured in the study.

In an initial experiment, we found that just 10 of 30 fearful dogs in the control group passed the SAFER test, whereas 23 of 30 fearful dogs in the treatment condition passed, a difference that was highly significant [100]. These results were replicated in a second experiment in which only 2 of 16 fearful control dogs and 15 of 16 fearful dogs receiving our enrichment passed the SAFER. For comparison, we also included groups of non-fearful dogs, nearly all of which passed the SAFER regardless of whether they received enrichment or not [100] (Table 2). While we certainly do not encourage those without appropriate qualifications to work with shelter dogs exhibiting fear-induced aggression, we do believe these results are an example of enrichment centered around human interaction buffering meaningful behavioral effects of stress in the shelter environment. They also highlight the value of attending to individual differences in designing treatment strategies. In addition, they align with conclusions of others [103] advocating for the discontinuation of temperament tests for testing adoption suitability (as has been done in the shelter in which we conducted our work).

## 4. Conclusions

Lasting behavioral consequences of stress exposure in translational laboratory experiments or in the dog shelter are rightfully considered to be negative outcomes if they serve as models of human suffering or pathology, or in the case of the shelter, reduce welfare or the likelihood of successful adoption. Yet many such outcomes appear to have derived from behaviors that were adaptive in natural environments. If stressful conditions experienced by a young animal, or by its mother prenatally, are an indication that conditions are likely to be stressful in that environment as the individual matures, it can be beneficial for that animal to alter its development to best address the expected future environment. Thus, behavioral plasticity, which can allow for changes in the developmental trajectory to better suit the predicted environment—whether plasticity is induced by glucocorticoids or by other means—will be subject to natural selection [104,105]. This notion of “predictive adaptive responses” is thought to describe a common evolutionary process [76,106,107,108]. In a stressful, competitive environment, behavioral traits such as reduced sociality, increased reactivity and aggression, and a re-focusing of cognition on skills relevant to basic survival at the expense of “higher level” skills might all be adaptive and, indeed, all have been documented to develop disproportionally following early stressful conditions [108,109,110]. While specific behavioral outcomes vary by species and situation, there is no reason to expect dogs to be exempt from these influences. We cannot be sure that the stress of shelter exposure or the glucocorticoid elevations or other stress-related physiological changes induced by stress encountered in the shelter are sufficient to produce such changes in behavior development. However, this remains a possibility, particularly for dogs that are especially sensitive to stressors. The concept of predictive adaptive responses might afford a useful perspective from which to consider stress in the shelter and its outcomes in future studies. From a practical point of view, the chance that stressors encountered in the shelter may be shaping later behavior in unwanted ways reinforces continued efforts to reduce shelter stress, such as reviewed here, even if a later negative behavioral or welfare outcome cannot be documented at the present time.

## Figures and Tables

**Table 1:** Relative preference for, and buffering by, specific titi monkey family members.

Subject	Preference for	Buffering by
Mother	Father > Infant	Father yes; Infant no
Father	Mother > Infant	Mother yes: Infant no
Infant	Father > Mother	Both yes, Father > Mother

Data from Mendoza and Mason [25] and Hoffman et al. [26].

**Table 2:** Number of fearful enrichment and control dogs that passed and failed the SAFER test in the first experiment of Willen et al. [100].

	Pass	Fail	% Pass
**Experiment 1**			
Fearful			
Enriched***	23	7	77
Control	10	20	33
**Experiment 2**			
Fearful			
Enriched***	15	1	94
Control	2	14	12
Non-Fearful			
Enriched***	14	2	87
Control	15	1	94

*** differs from total fearful control dogs, *p* < 0.001.
